# Quality care in vesico-vaginal obstetric fistula: case series report from the regional hospital of Maroua-Cameroon

**DOI:** 10.4314/pamj.v5i1.56192

**Published:** 2010-04-27

**Authors:** Pierre Marie Tebeu, Joseph Nelson Fomulu, Achille Aurelien Mbassi, Jean Marie Tcheliebou, Anderson Sama Doh, Charles Henry Rochat

**Affiliations:** 1 Ligue d’Initiative et de Recherche Active pour la Santé et l’Education de la Femme (LIRASEF); 2 Department of obstetrics and Gynecology, Provincial hospital, Maroua, Cameroon; 3 Department of obstetrics and Genecology, University hospitals, Yaoundé-Cameroon; 4 Geneva Foundation for medical Education and Research (GFMER), Geneva, Switzerland

**Keywords:** Obstetric fistula, closure, continence, Cameroon

## Abstract

The World Health Organization (WHO) proposes a successful closure rate for first repair of vesico-vaginal obstetric fistula to be at 85% in each facility, with the continence achievement among the closed cases at 90 %. We are reporting the vesico-vaginal obstetric fistula outcome at the provincial hospital of Maroua-Cameroon from 2005 to August 2007.  Among the overall 32 patients with vesico-vaginal fistula operated, 25 patients were at their first operation. The complete closure of vesico-vaginal fistula (VVF) was 23/25 (92%) and among the 23 patients with complete closure 17(74%) had good continence. When we consider only the 25 patients who were at their first operation, the overall closure of VVF was 23/25 (92%) and among them 17/23 (74%) were continent. Large lesion, bladder neck lesions, vaginal adherence and rigid margin are associated with failure/incontinence. These factors must be taken into consideration when preparing patients for surgery or when assigning them to a surgeon within the surgical team.

## Background

Some studies present the result of obstetric vesico-vaginal fistula (VVF) repair including repeating operations for the same patient [1]. Other studies present the result only as successful closure without taking into consideration the continence status [2]. WHO proposes the successful closure rate for first repair at 85% in each facility with the continence achievement among the closed cases at 90 % [3]. In a recent study, we reported the risk factors for obstetric fistula in far North Cameroon [4], however, little is known about the treatment outcome. The objective of this study was to analyze the vesico-vaginal obstetric fistula outcome at the Regional Hospital of Maroua-Cameroon.

## Methods

This was a case series study at the provincial hospital of Maroua-Cameroon from May 2005 to August 2007. The Maroua Provincial Hospital is a second level referral hospital for the Far North Province of Cameroon.

Women consulted in the Maroua provincial hospital for leakage of urine or stools through the vagina. The site and, size of fistulas were confirmed by speculum examination. Transurethral catheter was performed and followed by instillation of 1% methylen blue in the bladder to trace the leakage in small cases. During the study period we identified 48 patients with genital fistulae. We excluded the non obstetrical fistulas (n=6); obstetrical recto-vaginal fistula (n= 3) as the study focused on vesico-vaginal fistulas; and obstetric VVF fistula treated by diversion (n= 7). Finally 32 obstetrical VVF were analyzed.

The Surgical technique included an infiltration with adrenaline serum, an incision in V/Y, a vésico-vaginal division and a suture without tension in two layers with monofilament and semi-resorbable thread. The V/C incision was done for fistula localized on the uterine cervix. Data were collected on the socio-economic status (age, marital status, educational level, occupation), reproductive history; circumstance of the occurrence of obstetric fistula; anatomic characteristics of the fistula and the treatment outcome.

The data were collected in the standard file prepared for obstetric fistula patients. The information from the file was entered in the Excel software by an assistant nurse with good computer skills. Data was stored in the obstetric fistula database of the Department of Obstetrics and Gynecology of the Maroua provincial hospital. The data of this study was retrieved from the obstetric fistula database of the Department of Obstetrics and Gynecology in the provincial hospital of Maroua-Cameroon between May 2005 and August 2007.

Data analysis was performed using EPI Info 3.4. This descriptive analysis includes mean, median, range, standard deviation, 25% quartile (q1), 75% quartile (q3), and proportions. The success rate was calculated as proportion of cure among treated patients. Characteristics from patients who were treated successfully were compared with patients with failure (i.e. not closed fistulas and residual incontinence).

## Results

The mean age of the 32 patients at the time of intervention was 36.2 (SD: 14.3) years; 30(94%) of women had no occupation; 7(22%) were divorced; 4(12.5%) were widows and 28(88%) had no formal education; 18(56.3%) patients did not have antenatal care during the indexed pregnancy; 27(84.4%) of patients had been in labor for more than 12 hours and 28 (87.5%) pregnancies resulted in stillbirth (Table 1).

The patient’s duration of stay with obstetric fistula ranged from 1 month to 33 years, with the median duration at 8.5 years. Among the 32 patients with obstetric VVF, 18 (56%) were at bladder neck region, 26 (81%) were more than 2 cm of size, and 22 (68.8%) had vaginal adhesions (Table 2).

Among the overall 32 patients with vesico-vaginal fistula, closure was observed in 30 (93.5%) patients. The complete closure with good continence was observed in 22 (73.3%) of the 30 patients; closure with residual incontinence observed in 8(26.7%) of the 30 patients repaired and failure of closure was observed in 2 out of the 32 patients (6.2%) patients.

Among the overall 32 patients with vesico-vaginal fistula operated, 25 patients were at their first operation. The complete closure of vesico-vaginal fistula was 23/25 (92%) and among the 23 patients with complete closure 17(74%) had good continence. The continence procedure was performed for two patients with the transobturator technique (TOT), but only one of them had a good continence.

We compared the overall 22 patients with closure and good continence mentioned above with the 10 patients with failure/incontinence. We found that patients with failure/incontinence have fistula more likely to be localized at the bladder neck region (80% vs. 50%); with size more than 5 cm (30% vs. 9%); with vaginal adherence (80% vs. 64%) and rigid edges (80% vs. 55%) (Table 3).

**Table 1:** Clinical manifestations in patients with the obstetric fistula

**Characteristics**	**Frequency among vesico vaginal fistula cases (N=32)**	
		**n**	**%**
**Vulva irritability**		
	No	22	68.8
	Yes	10	31.2
**Vaginal itching**		
	No	31	96.9
	Yes	1	3.1
**Dysuria**		
	No	29	90.6
	Yes	3	9.4
**Difficulties of walking**		
	No	29	90.6
	Yes	3	9.4
**Urine lose**		
	No	0	0
	Yes	32	100
**Vulvar Dermatitis**		
	No	15	46.9
	Yes	17	53.1
**Height**		
	147-149	2	6.2
	150-167	15	46.9
	unknown	15	46.9
**Leucorrhoea**		
	No	30	93.8
	Yes	2	6..2
**Inflammatory cervix**		
	No	27	84.3
	Yes	2	6.3
	Not seen	3	9.4

Median duration of obstetric fistula was 8.5 years (Q1=0.8yrs; Q3= 20.5yrs)

**Table 2:** Characteristics of fistula lesions

		**Vesico-vaginal fistula (N=32)**	
Characteristics	**n**	**%**
**Fistula palpable**		
No	0	0
Yes	32	100
**Fistula edge**		
Soft	12	37.5
Rigid	20	62.5
**Localization**		
Bladder	5	15.6
Trigonal	9	28.1
Cervical	10	31.3
Urethral	8	25.0
**Fistula size**		
< 2 cm	6	18.8
2-5 cm	21	65.7
<5 cm	5	15.5
**Vaginal adherence**		
No	10	31.2
Yes	22	68.8
**Surgical approach**		
Vaginal	27	84.4
Abdominal	2	6.3
Combined	3	9.4
**Complementary maneuver at surgery**		
None	5	15.6
Martius	24	75.0
Epiloon interposition	1	3.1
Re-implantation	2	6.3
**Duration (in minutes) of operation, median (percentile 25 and 75)**		145 (115;195)
**Duration (in days)of bladder draining, median(percentile 25 and 75)**		12 (11;14)
**Surgical outcome**		
Close and continent	22	68.8
Close but not continent	8	25.0
Complete failure	2	6.2

n=number of cases; %=percentage

**Table 3:** Comparison of the patients with closure and continence and those with failure/incontinence

Characteristics	Obstetric fistula			
		Success (N=22)		Failure/incontinence (N=10)	
		n	(%)	n	(%)
Edges				
	Soft	10	45.5	2	20
	Rigid	12	54,5	8	80
Localization				
	Bladder/trigon	11	50	2	20
	Cervico-urethral	11	50	8	80
Fistula size				
	=< 5 cm	20	90.9	7	70
	6-10	2	9.1	3	30
Vaginal adherence				
	No	8	36.4	2	20
	Yes	14	63.6	8	80

## Discussion

This study is the first according to our knowledge presenting the quality care in vesico-vaginal obstetric fistula according to the recent WHO recommendations. Among the 32 patients with VVF, the overall closure was observed in 30 (93.5%) cases. Our result fit in the WHO target of 85% [3]. In the literature the overall closure range between 81-100% [5]. However, the overall closure does not necessary mean that the patient is cured, as 7-23% of the treated patients will have closure of their fistula, but still with a residual incontinence [5]. In the present report 8 out of the 32 patients (25%) had closure, but still with residual incontinence. The continence procedure performed for two patients with TOT, but only one of them had a good continence. This minimally invasive operation should be further assessed so that it can demonstrate its effectiveness and probably become the "Gold standard". We suggest that the procedure should be evaluated in all fistula sites to improve their continence rate.

When we consider only the 25 patients who were at their first operation, the overall closure of VVF was 23/25 (92%) and among them 17/23 (74%) were continent. WHO recommends that 90% of the closed fistula should be also continent, and this implies the complete cure of the patient [3]. In the present study, 17 out of the 23 patients at their first attempt of repair were continent (74%). The high rate of residual incontinence observed in this study could be due to the deteriorated anatomic presentation of our patients at surgery. Previous studies found that size of the fistula hole of more than 5cm, vaginal adherence and rigid margins were associated with poor surgical outcome [1]. The size of the sample is likely too small to determine differences between the groups with 'successful' outcomes versus those with failure/incontinence with any significance. We strongly recommend more studies with big sample for comparison between the two groups. Patient characteristics like parity and body stature are also of interest; however they are risk factors of obstetric fistula occurrence rather than those of quality care [6, 7].

## Conclusion

Large lesion, bladder neck localization, vaginal adherence and rigid margin are associated with failure/incontinence. These factors must be taken into consideration when preparing patients for surgery or when assigning them to a surgeon within the surgical team.

## Competing interests

There are no competing interests in relation to this manuscript.

## Author’s contribution

PMT: study concept, design, data collection, statistical analysis, manuscript writing, and review. JNF: manuscript writing and review. AAM: data collection. JMT: manuscript writing, and review. ASD: manuscript writing, and review. CHR: study concept, design, manuscript writing, and review.
